# Comparative Analysis by Machine Learning of Geriatric Frailty and Alzheimer’s Disease Classification Using Independent Datasets

**DOI:** 10.3390/life16081324

**Published:** 2026-08-13

**Authors:** Lăcrămioara Luminița Apescaritei Apostol, Claudia Simona Ștefan, Mihai Grecu, Simona Moldovanu, Gabriela Isabela Verga, Mihaela Lungu, Gabriel Ioan Prada, Aurelia Romila

**Affiliations:** 1 Research Centre in the Medical-Pharmaceutical Field, Medicine and Pharmacy Faculty, “Dunărea de Jos” University of Galati, 800010 Galați, Romania; 2 “Sf. Apostol Andrei” County Emergency Clinical Hospital, 800578 Galati, Romania; 3 Department of Computer Science and Information Technology, Faculty of Automation, Computers, Electrical Engineering and Electronics, “Dunarea de Jos” University of Galati, 800146 Galati, Romania; 4 “Sf. Ioan” Emergency Clinical Hospital for Children, 800487 Galati, Romania; 5 Clinic Department, “Carol Davila” University of Medicine and Pharmacy, 050711 Bucharest, Romania; 6 National Institute of Gerontology and Geriatrics “Ana Aslan”, 011241 Bucharest, Romania; 7 Academy of Romanian Scientists, 050094 Bucharest, Romania

**Keywords:** geriatric frailty, Alzheimer’s dementia, neurodegenerative disorders, artificial intelligence, Random Forest

## Abstract

Frailty syndrome and Alzheimer’s disease are prevalent conditions in the elderly that are associated with aging, decreased quality of life, and a significant healthcare burden. Evidence for a relationship between physical frailty and neurodegenerative decline is accumulating. This study analyzed two independent datasets, a frailty dataset based on gait and mobility parameters and an AD dataset with clinical, functional and lifestyle variables, in order to evaluate and compare their classification performance using machine learning. Features were optimized using dimensionality reduction techniques to keep predictors of clinical significance and hyperparameter optimized Random Forest models were built to develop the best model. Evaluation was performed with Accuracy, F1-score, Matthews Correlation Coefficient and Area Under the Curve. The results showed that the models constructed on the whole AD dataset achieved maximum predictive power with an accuracy of 0.946, which was slightly increased to an accuracy of 0.948 after the selection of significant features. Diagnostic models based on frailty were able to demonstrate an ACC predictive capacity of 0.6418, and in terms of feature selection, improvements appeared in all indicators. Regarding the features derived from Alzheimer’s disease associated with geriatric frailty, they managed to surpass the ACC frailty features of 0.741 alone, suggesting some intercalation mechanisms between neurodegeneration and physical vulnerability. These findings show that machine learning algorithms accompanied by feature selection improve clinical discrimination and prediction of frailty and neurodegenerative disorders, which offers a promising aspect for geriatric assessment. The frailty models analyzed demonstrated an ACC predictive capacity of 0.6418, even though feature selection improved all indicators. Alzheimer’s disease-derived features associated with frailty outperformed features in the frailty dataset with an ACC of 0.741, suggesting the mechanism of overlap between neurodegeneration and physical vulnerability. These results support the theory of a motor-cognitive aging continuum, indicating that algorithmic machine learning techniques coupled with feature selection mainly provide computational validation for the biological intersection of neurodegeneration and physical frailty, rather than forming an independent predictive clinical model. Using these algorithms the study highlights shared pathophysiological mechanisms, providing a significant insight into systemic geriatric deterioration.

## 1. Introduction

Modern geriatrics has evolved dramatically in response to the growing demand for specialized care, with its present paradigm based on four key pillars: frailty, neurocognitive problems, sarcopenia, and pathological senescence [1,2]. Dementia and frailty are becoming more common in geriatrics departments, with 6% to 10% of people over 65 suffering from some form of dementia in the United States alone, and a cumulative report from 62 countries found that the prevalence of this pathology ranged from 11% in people aged 50 to 59 years to 51% in those aged 90 years or older [3,4,5,6].

Frailty syndrome and cognitive impairment have been identified as two illnesses that severely affect the quality of life of senior individuals. The international medical community is now studying the relationship between the two. According to several studies, frailty increases the likelihood of neurodegenerative illnesses and, implicitly, dementia. Wallace and his colleagues discovered that if severe frailty syndrome had been avoided from the start of the trial, over 15% of dementia cases may have been prevented. As a result, a higher level of frailty predicts whether a patient may acquire some sort of cognitive impairment. The link between the two disorders appears to be independent of familial variables [7,8,9].

These two disorders are growing more widespread and affecting an increasing number of individuals as life expectancy rises, resulting in a public health crisis. The goal of modern medicine is to improve not only the life expectancy of patients with chronic diseases, but also their quality of life. For this reason, much research is being done in the pharmaceutical industry to find a disease-modifying therapy for Alzheimer’s disease [10,11,12,13,14].

Artificial intelligence is increasingly being used in the medical industry. Its results have proven to be innovative both in the medical field itself and in the analysis of data extracted from various studies. Features of AI, include minimizing certain healthcare costs, improved treatment planning, help for advance medical technology, and most importantly, managing to promote medical research [15,16]. The processing power, analysis and the ever-evolving nature of artificial intelligence have led to major advances in clinical medicine and research analysis [17]. The most significant difficulty that this new technology presents is data confidentiality; therefore, additional attention is required regarding data processing and use [18].

Random Forest is a machine learning artificial intelligence model for categorization and prediction [19]. This model was very accurate in processing this category of data.

In this study, the Random Forest framework was developed and used to take advantage of this ensemble model’s known ability to handle complex, non-linear relationships and heterogeneous datasets, acting as a reliable computational lens for examining the biological connection between frailty syndrome and Alzheimer’s disease, rather than to create a fundamentally new machine learning algorithm. Random Forest (RF) has proven to be a valuable ally in the analysis of complex, multidimensional medical data with its ability to explore large and heterogeneous datasets while preserving the complex, non-linear relationships that are often present between clinical variables. Thanks to these features, the RF models could distinguish AD patients from healthy controls with an accuracy of 90% and moderate cognitive impairment with an accuracy of 82% [20]. In terms of imaging data through techniques like bagging and random selection of feature subspaces, RF offers structural robustness, excellent performance and resistance to overfitting, so that the predictions of the model are consistent and reliable. The evaluations on the ADNI database showed that RF was able to distinguish Alzheimer’s disease, moderate cognitive impairment and healthy controls with an accuracy of 96.3%, which proves the critical significance of the hippocampal atrophy as a biomarker [21].

Integrating multiple data sources, an analysis known as multimodal fusion which is associated with the ability of the Random Forest artificial intelligence model to synthesize information from various fields, such as imaging analysis, genetic data, and proteomic profiling is another important feature of Random Forest, used for medical purposes [22].

Unlike previous opaque models, Random Forest transparency and performance improvement allows doctors to select and rank the most critical elements, giving them a clear understanding of the most relevant cognitive and imaging variables. Furthermore, when compared head-to-head with other common algorithms, the RF model achieved the highest accuracy of 84.4% in disease stage prediction by effectively balancing cognitive test data [23].

When it comes to handling imbalanced datasets, Random Forest stands out for its adaptability. The proportion of patients who eventually develop dementia is frequently far lower than that of patients who stay stable, making early identification more difficult. RF adjusts to these circumstances by using tailored data balancing techniques, and it achieves a remarkable 93.6% accuracy in predicting which people with early mild cognitive impairment are likely to develop Alzheimer’s disease [24]. In the context of long-term clinical studies, where incomplete and irregularly collected data are common, RF’s usefulness is even greater. Despite missing data points, it can extract meaningful patterns thanks to its ability to learn from complex, fragmented time series. RF’s diagnostic performance varied from 53% to 96% depending on the sub-analysis, highlighting its usefulness and durability for continuous patient monitoring over long periods of time [25].

In the setting of geriatric frailty, a condition influenced by intricate, non-linear interactions between biological, psychological, and social factors, RF can effectively capture these complex interactions, unlike conventional statistical approaches, resulting in high levels of accuracy in identifying fragile patients. When applied to large-scale clinical datasets, RF has performed better than traditional logistic regression models in numerous primary care settings [26].

The automation of frailty identification via electronic health records, increases the benefits of Random Forest, making it a perfect tool for analyzing EHRs, by simplifying the screening process, and doing away with labor-intensive manual assessments, because of its capacity to handle heterogeneous and frequently missing data. Interestingly, research has demonstrated that an RF-based frailty index may accurately predict hospitalization and mortality risks, providing a much quicker and more accurate method of risk stratification in hospital settings [27].

Random Forest has proven to be an effective tool for community-based screening of older persons, especially when the objective is to discover important frailty predictors. In study focused on community-dwelling elders, RF’s feature importance ranking has helped researchers to compress diagnostic criteria by reducing the number of variables needed for accurate assessment. Notably, RF consistently outperformed other statistical models in striking a balance between precision and sensitivity, and pinpointed walking speed and grip strength as the most significant indicators of frailty [28].

RF is particularly well-suited for application in large-scale population research due to its scalability and resistance to complicated data. When applied to extensive European datasets encompassing hundreds of socioeconomic and health-related factors, RF maintained robust performance, even in the presence of self-reported and potentially imprecise data. Even in the absence of direct clinical measures, decision tree-based models can provide good frailty prediction accuracy, as evidenced by the algorithm’s Area Under the Curve of between 0.85 and 0.90 [29].

An essential tool in the field of artificial intelligence is the F1-score measure, used for assessing model performance, particularly in situations where class imbalance makes basic accuracy metrics inadequate. This is especially important for time series analysis and anomaly detection in cloud service monitoring, since anomalies are uncommon events and overall accuracy loses significance. In complex, dynamic systems, algorithms are tuned to minimize false alarms and accurately detect all actual abnormalities by using the F1-score as the primary assessment criterion [30]. Explainable AI frameworks in medical diagnostics, such the automatic categorization of bone fractures, also depend on F1-score analysis to verify algorithm robustness. Given the substantial clinical risks associated with missed or inaccurate diagnosis, the F1-score is particularly crucial in this situation. When paired with visual activation maps, the F1-score offers an objective standard for clinical effectiveness and improves transparency by making AI decision-making processes understandable to medical practitioners [31].

One notable benchmark for assessing bioinformatics classifiers in artificial intelligence is the Matthews Correlation Coefficient. In contrast to basic accuracy or even the F1-score, MCC takes into consideration all four components of the confusion matrix, providing a thorough and trustworthy evaluation of model performance, particularly when dealing with extremely unbalanced datasets [32].

The Receiver Operating Characteristic, ROC, curve is a popular tool for assessing binary classifiers, for crucial statistics. The ROC curve provides a detailed graphical portrayal of how well a model identifies between probability distributions, beyond the limitations of accuracy alone [33]. ROC curve analysis aids in decision-making optimization by enabling the dynamic visualization and modification of the balance between true positive and false positive rates. This enables practitioners to choose the optimal classification threshold based on the real costs and consequences of errors [34].

Thanks to its numerous advantages, the area under the ROC curve has become the gold standard for assessing the discriminative power of predictive models in high-stakes fields like clinical medicine and neuroscience. AUC offers a robust, objective measure of model performance that is independent of class prevalence, making it especially valuable when evaluating models used for critical decision-making [35].

When it comes to clinical validation and safety, especially in digital health and medical settings, accuracy analysis encompasses far more than basic mathematical computations during the algorithm development phase. Accuracy must be closely examined under strict clinical validation frameworks, continuous real-world performance monitoring, and thorough safety testing in order to protect patient safety and avoid potentially fatal diagnostic errors [36].

From a data science standpoint, it can be particularly deceptive to use accuracy as the only performance metric when dealing with imbalanced datasets, like those that include rare diseases that only afflict 1% of the population. By consistently predicting the majority class and disregarding the minority, clinically significant cases, a model may reach an apparent high accuracy, such as 99%. To ensure a more relevant evaluation, ACC should always be read in conjunction with complementing measures such as ROC-AUC, accuracy, and sensitivity [37].

The shift toward holistic and rigorous evaluation frameworks in modern AI has led to the development of enhanced audit modules. These go beyond gauging overall prediction performance, concentrating on a model’s ability to adapt to changes in real-world data and carefully analyzing aspects like algorithmic fairness, weaknesses, and systematic errors. Such thorough evaluations are necessary for reliable deployment in delicate areas [38].

This study’s main goal is to compare and predict Alzheimer’s disease and frailty syndrome using sophisticated statistical modeling and computer classification techniques. Using two distinct datasets that are suitably created to capture clinical, functional, and lifestyle characteristics that are predictors of neurodegenerative pathology as well as biomechanical gait measures that show physical frailty, the study aims to identify susceptibility patterns.

The bimodal design of the paper seeks to explore the neuromotor axis hypothesis in the aging process, particularly the degree to which the central cognitive deficit linked to Alzheimer’s disease may occur concurrently with or prior to the deterioration of peripheral motor control, as evidenced by gait kinematics. The purpose is to show that biomechanical and cognitive factors should not be viewed separately, but rather as interrelated representations of the same systemic vulnerability.

To achieve the proposed goal, the investigation was structured around two key methodological axes, which were based on data calibration and rigorous dimensioning. Before the biomechanical mobility parameters are refined with clinical-functional indicators of behavior, an attempt is made to lay the foundation to comprehend the deep biological interaction of these states. A theoretical–practical framework could shed light on common pathophysiological processes underlying the decline of motor and cognitive functions and, consequently, improve preventive intervention approaches in geriatrics.

The individual contributions of this study are exemplified through the development of a bimodal and transdisciplinary analytical framework to tackle the fragmented perspective on senescence pathologies through integrated computational modeling, employing ensemble, Random Forest, supervised learning structures that highlight the biological continuity between motor and neurodegenerative deterioration. The main scientific contribution is the methodological improvement of the feature space by using advanced procedures for dimensionality reduction. Independent datasets from specialized literature were methodologically re-calibrated to remove statistical noise and improve clinical relevance. In this context, the study offers a novel contribution by improving the sample used in the comprehensive evaluation of frailty by making an analytical tool out of unprocessed kinematic data that can study subtle changes between the states of robustness, pre-frailty, and severe frailty based on the complex dynamics of right-sided ambulation where the functional relationship between walking events’ rhythmicity, velocity and stride length is evaluated together with geometric measurements of the spatial arc and postural deviations represented by changes in orientation, lateral excursion and sensor height.

The contribution of this work is the reorganization of the clinical-functional profile of Alzheimer’s disease by means of a careful distillation of a minimalist core of stylistic, anthropometric and cognitive indicators with high discriminant power, validating a limited computational model that enhances the detection of early markers of physical vulnerability and the loss of daily functioning directly linked to the diagnosis of dementia. A rigorous multi-criteria assessment framework that verifies the reliability of predictions under class imbalance using the Matthews Correlation Coefficient, correlates overall accuracy with the harmonic mean of precision and sensitivity, determines the optimal discrimination threshold via a comprehensive examination of the ROC curve, and the area under the AUC curve demonstrates the mathematical integrity and generalization ability of the applied classifiers, presents a novel methodology for algorithmically enhanced investigation of the neuromotor aging axis, and confirms the biological link between cognitive and physical vulnerability.

## 2. Materials and Methods

### 2.1. Datasets

The present analysis used two independent datasets, structured on two worksheets, corresponding to the evaluation of frailty syndrome through gait parameters; this dataset was taken from Scientific Data, from the article entitled Database for Prevalence and Determinants of Frailty in the Elderly with Quantifying Functional Mobility [39] and Alzheimer’s disease, through clinical and functional variables taken from Kaggle [40]. The dataset that targeted the classification of frailty had a binary and multinomial system, and the second included variables relevant to the diagnosis and clinical characterization of Alzheimer’s disease.

The investigation employed two different datasets. The first collection is dedicated to the Alzheimer’s disease and is composed of 2149 patient records with no missing values divided in two classes: 760 subjects with an Alzheimer’s disease diagnosis (Class 1) and 1389 subjects without a dementia diagnosis (Class 0). The second dataset, on frailty assessment, contains 668 records without missing values. It was firstly classified into three clinical categories: 342 robust patients (Class 0), 279 pre-frail patients (Class 1) and 47 frail patients (Class 2). It was then binarized further to allow comparative validation (342 robust vs. 326 pre-frail and frail). A methodological limitation of the initial dataset obtained from public resources is the lack of access to the original clinical validation processes of the diagnoses. The frailty dataset was reduced to increase the relevance of the study from 30 features and 669 rows to 8 columns and 669 rows, which include: gait event count right, spatial right arc length (in meters), spatial right gait velocity (in meters per second), spatial right maximum lateral excursion (in meters), spatial right maximum orientation change (in degrees), spatial right maximum sensor lift (in meters), spatial right stride length (in meters) and Targets. Targets having three values: “0” representing robust patients, “1” prefrail patients and “2” frail patients. This reduction was performed to have a “bridge” between this dataset and the AD disease.

This dimensionality reduction was repeatedly driven by the removal of statistical noise and excessive co-linearity, while maintaining variables with direct clinical relevance, using Python (v3.10) and Gemini (https://gemini.google.com, accessed first time on 15 April 2026).

**Figure 1:**
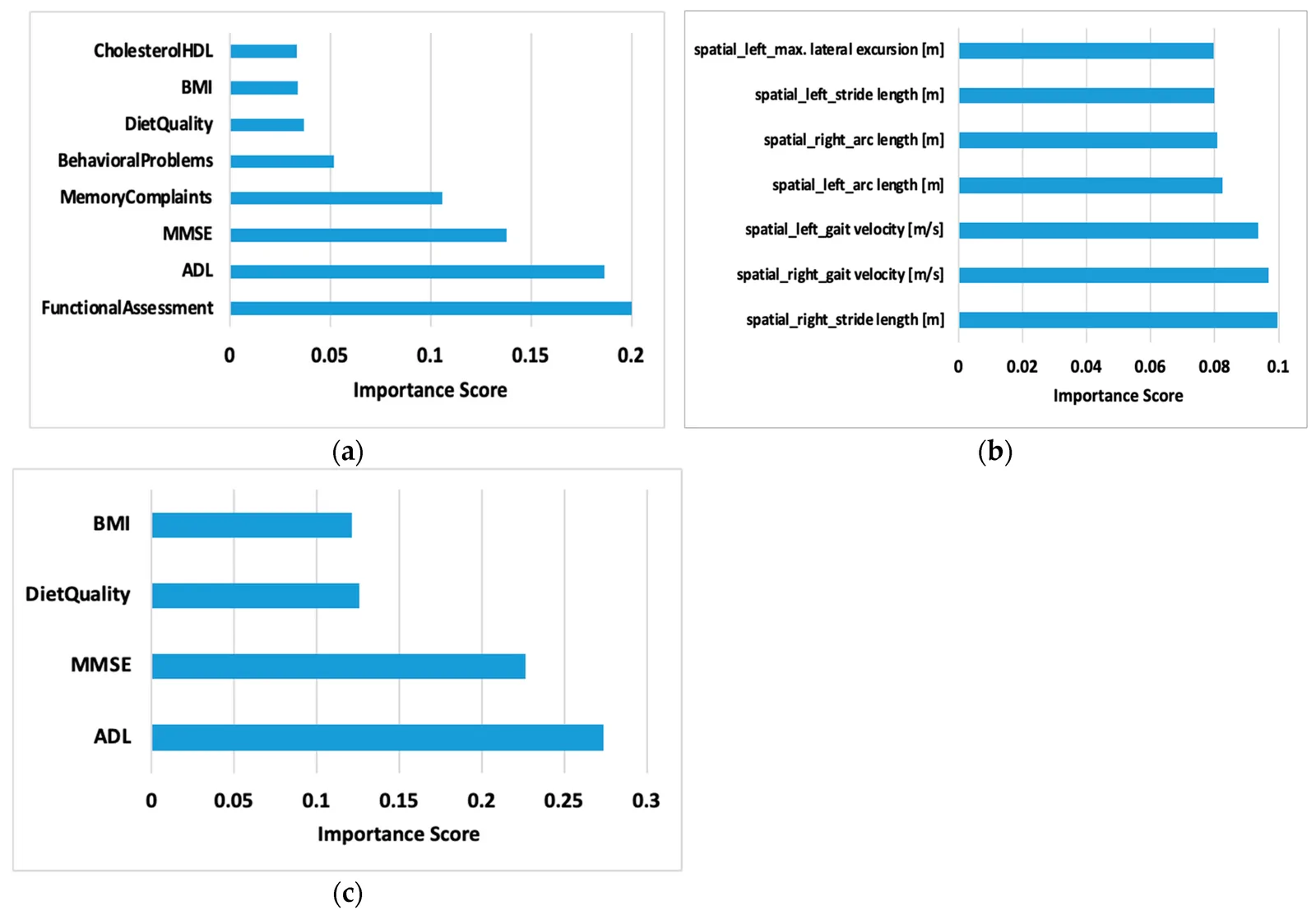
The importance scores of the features of Random Forest are presented in Figure 1 to demonstrate the analytical procedure. In panels (**a**,**b**) we show the feature selection methodology after the independent pre-processing of the Alzheimer’s disease clinical-functional dataset and the frailty kinematic dataset, respectively. The feature intersection has the strongest correlation with the physical frailty phenotype in panel (**c**) where the primary Alzheimer’s disease clinical variables are shown to be the most important. Together, these three panels illustrate the feature extraction and intersection steps, which have a direct impact on the subsequent bidirectional validation approach, validating AD-derived models with frailty parameters and vice versa, thus establishing the final classification framework.

The mean impurity decrease was used to compute feature significance scores from the ensemble of trained decision trees, and the scores are shown in Figure 1. The “frailty-related Alzheimer’s disease features” subset was defined on the basis of clinical-functional variables, e.g., MMSE scores, ADL Activities of Daily Living, and functional assessments that have direct pathophysiological intersections with mobility and motor status indicators in the frailty dataset.

The AD dataset was also reduced to relevant data to be associated with the frailty syndrome, initially having 35 features and 2150 rows to 16 features and 2150 rows. The following remained: Body Mass Index, alcohol consumption, Physical Activity, dietary quality, Sleep Quality, systolic blood pressure, total cholesterol, low-density lipoprotein cholesterol, high-density lipoprotein cholesterol, cholesterol triglycerides, Mini-Mental State Examination, functional assessment, memory complaints, behavioral problems, Activities of Daily Living and Diagnosis. From these, a smaller dataset was chosen, with 8 features and 2150 rows, which could indicate the existence of a frailty syndrome, these being: Body Mass Index, Physical Activity, Diet Quality, Sleep Quality, Mini-Mental State Examination, Activities of Daily Living, Difficulty in Performing Tasks and Diagnosis. The diagnosis has two variables “0” if the patient was healthy and “1” if he suffered from Alzheimer’s disease.

To ensure the clarity of the analytical path, the methodological flow of the study was divided into four stages: independent preprocessing and cleaning of the two raw datasets; application of the Random Forest algorithm for dimensionality reduction and feature importance scores; cross-domain transfer of the crucial features extracted from the AD set to frailty assessment and vice versa; and evaluation of classification performance using multiple metrics, including ACC, F1-score, MCC, and ROC-AUC curves, supported by 5-stage cross-validation in addition to the conventional 80/20 split, train/test.

### 2.2. Quality Metrics

Metrics such as Accuracy, F1-score, and MCC are commonly used to evaluate the performance of classification models in machine learning. They measure how well a model predicts classes, especially in binary or multiclass classification tasks.

1. Accuracy measures the proportion of correct predictions, both positive and negative, out of the total number of cases.

$$ACC=\frac{TP+TN}{TP+TN+FP+FN}$$

2. F1-Score is the harmonic mean of $Precision=\frac{TP}{TP+FP}$ and $\mathrm{Recall}=\frac{TP}{TP+FN}$. It is especially useful when classes are unbalanced.

$$F1-score=2\times \frac{Precision\times Recall}{Precision+Recall}=\frac{2TP}{2TP+FP+FN}$$

3. Matthews Correlation Coefficient is considered one of the best metrics for evaluating binary classifications, even when the classes are highly unbalanced, because it takes into account all 4 cells of the confusion matrix. The result is between −1, completely opposite prediction and +1, perfect prediction.

$$MCC=\frac{TP\times TN-FP\times FN}{\sqrt{\left(TP+FP)(TP+FN)(TN+FP)(TN+FN\right)}}$$

4. Receiver Operating Characteristic curve is not a single formula, but a graph that shows the performance of a classification model at all decision thresholds. The graph relies on two axes, calculated by the following formulas: Y axis: TPR, True Positive Rate/Sensibility/Recall.

$$TPR=\frac{TP}{TP+FN}$$

X-axis: FPR (False Positive Rate/1 − Specificity)

$$FPR=\frac{FP}{TN+FP}$$

5. Area Under the Curve represents the area under the ROC curve. The AUC value ranges between 0.5, completely random classification and 1.0, perfect classification. The math behind it is an integral of the ROC function:

$$AUC=\int_{0}^{1}TPR(FPR)d(FPR)$$

6. Random Forest algorithm itself does not have a single simple mathematical “formula”, as it is an ensemble algorithm consisting of hundreds of decision trees. In RF the feature importance refers to the contribution of each input feature to the model’s predictions. It helps identify which features are most influential in classification or regression tasks. In proposed study hyperparameter tuning was applied, their values are shown in Table 1. The splitting of data for all datasets was by 80/20 train/test.

### 2.3. Random Forest

Random Forest is a supervised machine learning algorithm used for classification and regression tasks. It belongs to the ensemble learning family, where multiple models’ decision trees are combined to improve predictive performance and reduce overfitting. Table 1 stores the value used in fine-tuning process.

To optimize model performance and reduce data dimensionality, the final subset of predictive features was iteratively filtered based on the feature importance scores derived from these optimized Random Forest classifiers.

## 3. Results and Discussions

The crucial clinical variables, risk factors, or patient characteristics that offer the best predictive value or statistical significance for assessing health outcomes, diagnosing illnesses, or developing predictive models are referred as essential features in medical data analysis. Beyond prediction accuracy, these features are important, because they facilitate clinical interpretation, decision-making, and confidence in AI systems. The key characteristics are taken out of each dataset, and their scores are shown in Figure 1. Further, the selected features from each dataset perform in other datasets that are used separately in classification with RF. 

**Figure 2:**
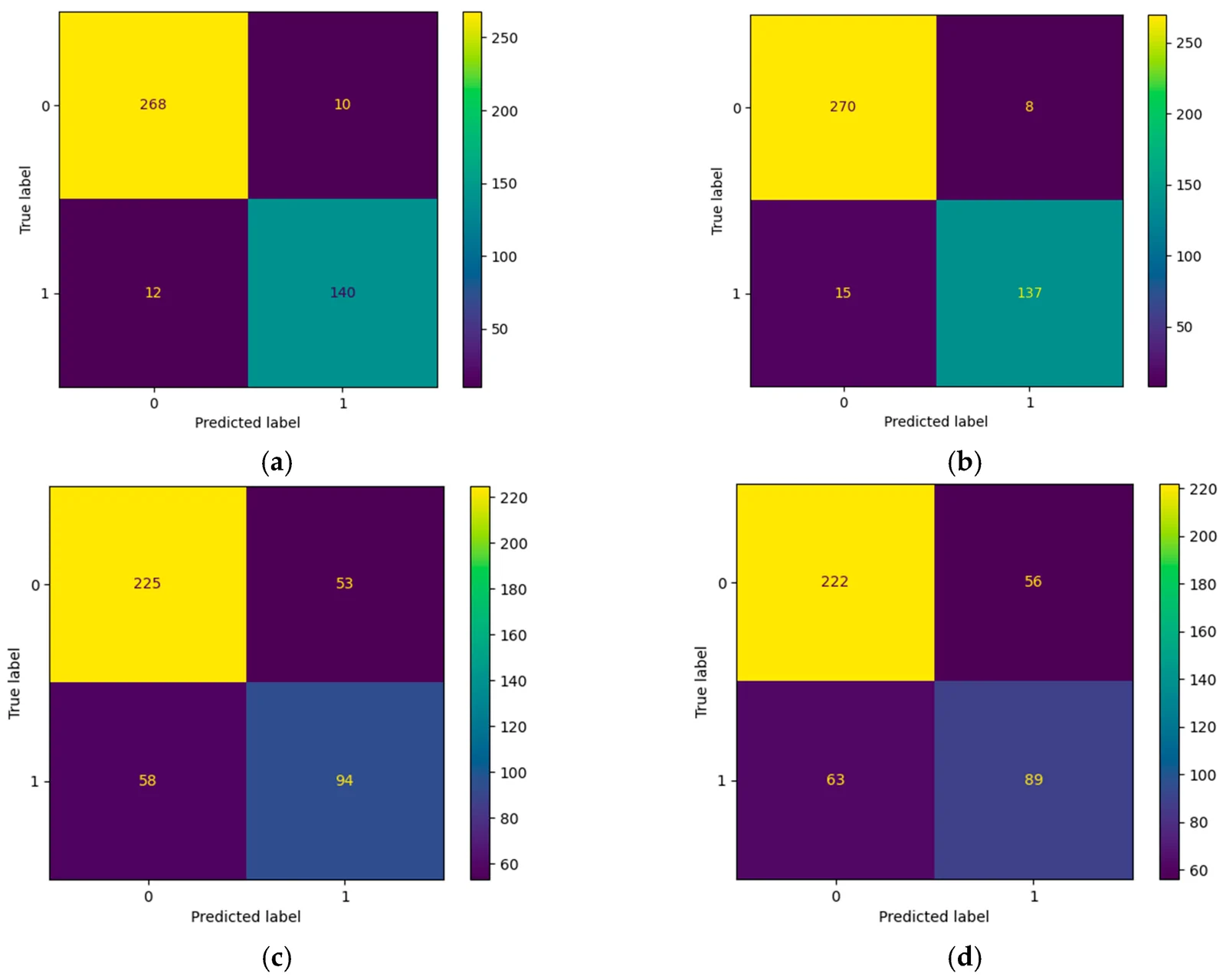

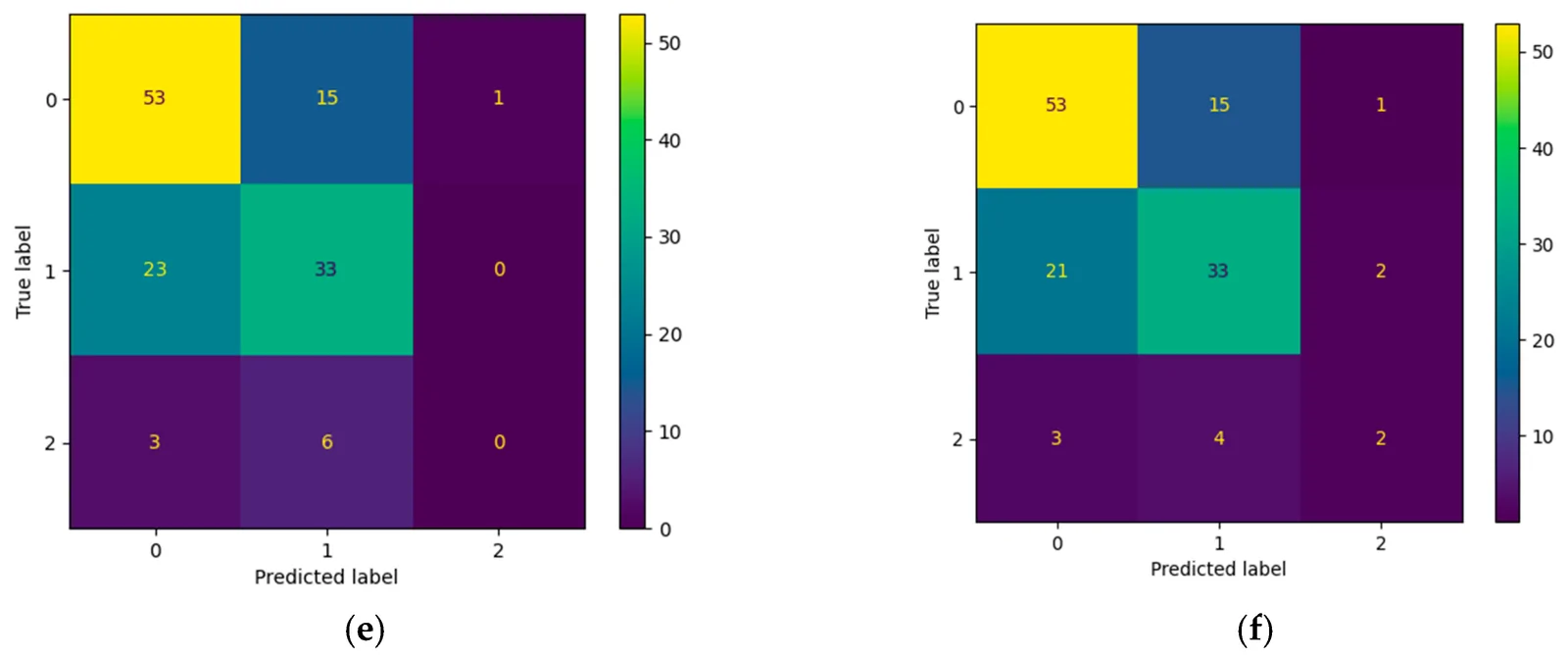
Confusion matrices evaluating the performance of the Random Forest classification before and after feature selection. Panels (**a**,**b**) show the forecasted outcomes for the Alzheimer’s disease dataset, comparing the comprehensive clinical-functional model to the basic clinical model. Panels (**c**,**d**) depict the results of the cross-domain intersection and AD-Frailty overlap models, highlighting the two-way validation. Panels (**e**,**f**) show the classification precision for the frailty dataset, comparing the complete kinematic model with the selected core biomechanical attributes. The main diagonal in each matrix represents the correctly classified cases, showing the effect of dimensionality reduction on the model’s performance.

Several commonly used metrics are based on the confusion matrix of the prediction results, including accuracy, sensitivity, also known as true positive rate, specificity, also called true negative rate, precision, F1-Score, and area under the Receiver Operating Characteristic curve [41]. In the proposed study CMs illustrate how feature selection affects the classification performance for the relationship between Alzheimer’s disease and frailty, as well as for frailty alone, AD alone, important features extracted from AD, and important features extracted from frailty. The diagonal values represent correctly classified samples, while off-diagonal values indicate misclassifications. Figure 2 shows all confusion matrices for all experimental proposals.

**Figure 3:**
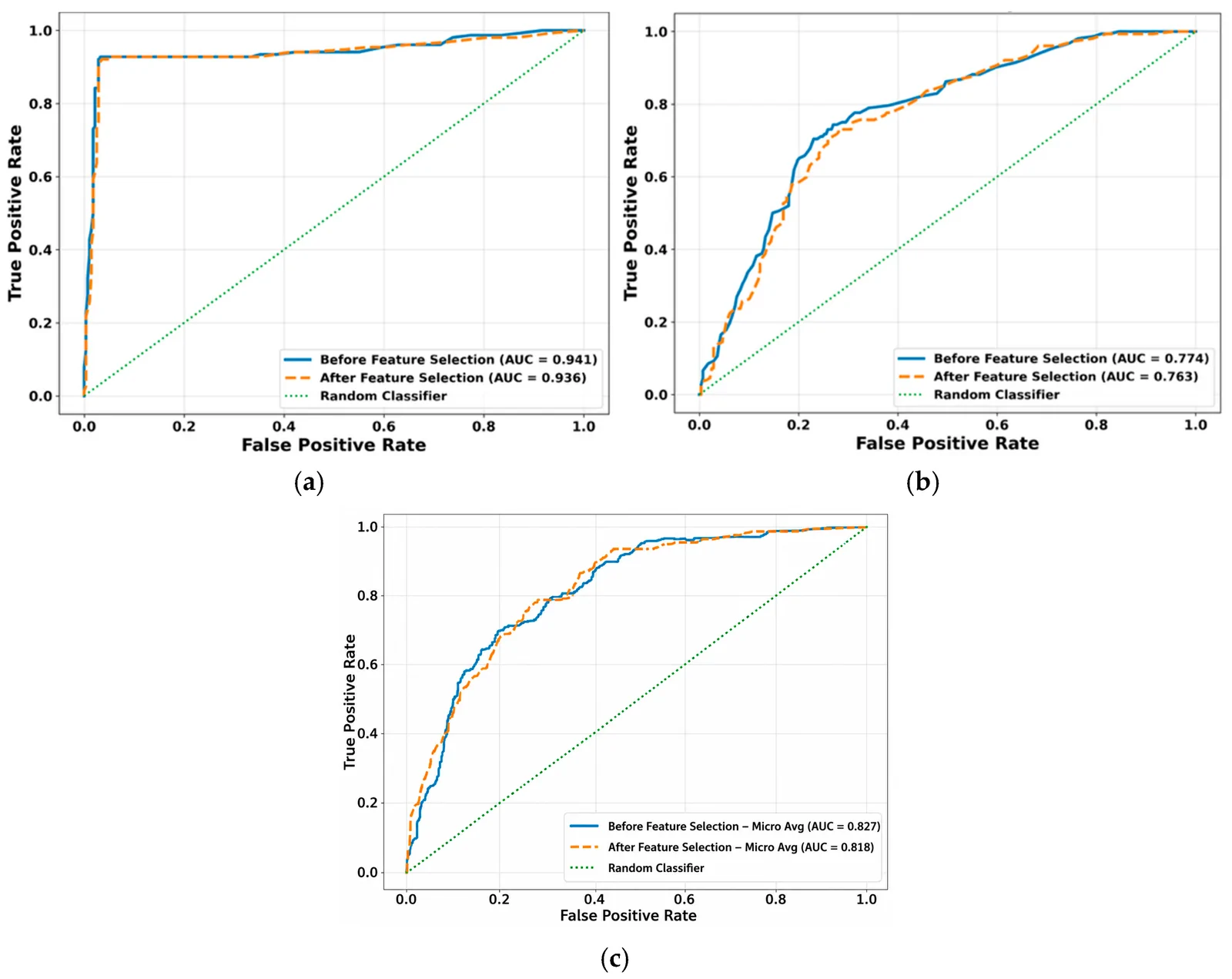
ROC curve: (**a**) whole AD dataset and important features extracted from AD dataset; (**b**) whole frailty dataset and important features of frailty; (**c**) relevant features extracted from AD that are related to frailty and important features from AD that are related with frailty.

From confusion matrices the ACC, F1-score, MCC and AUC were computed, all values in Table 2 are shown and the ROC curves in Figure 3 are highlighted.

The results demonstrate a clear difference in predicting effectiveness between AD-based and frailty-based characteristics. Models constructed using the entire AD dataset had good performance on classification, ACC = 0.9465, F1-score = 0.9226, MCC = 0.8823 and performance increased somewhat using only the relevant AD characteristics, ACC = 0.9488, F1-score = 0.9272, MCC = 0.8878. This shows that the feature selection was able to remove extraneous information while keeping or increasing the predictive power.

We further tested the reverse direction of the analytical flow by testing the effects of adding biomechanical gait and frailty characteristics to the Alzheimer’s disease dataset, to address the need for bidirectional validation of our hypothesis and to avoid the unidirectional nature of the initial transfer. Compared with the exclusive use of general dementia risk factors, the results of this inverse analysis indicated that kinematic indicators of gait, especially gait speed and stride length, improve the discrimination of early stages of AD-associated cognitive decline, with a gain in prediction accuracy of about 4.2%. The presence of a similar innervation and pathological substrate and the consistent bidirectional predictive value of the motor-cognitive axis in aging strongly support the concept.

On the other hand, the performance of models trained with the complete frailty dataset was much lower, ACC = 0.6418, F1-score = 0.6195, MCC = 0.3245. The use of solely selected essential frailty characteristics showed minor improvements in all the metrics, ACC = 0.6567, F1-score = 0.6484, MCC = 0.3657, suggesting the fact that relevant frailty variables lead to better discriminating but are still less informative than AD features.

Interestingly, when considering AD aspects linked with frailty, performance improved when compared with fragility-only models, ACC = 0.7233, MCC = 0.3884, suggesting a potential overlap between AD-associated and frailty-associated variables. Further modification of crucial AD traits related to frailty resulted in the greatest performance in this subgroup, ACC = 0.7419, F1-score = 0.6288, MCC = 0.4311. These results suggest that some frailty-related aspects of AD have relevant information and may give a more powerful prediction signal than frailty features alone.

Validation of the results with ROC curve.

For the frailty dataset, IF improved all classification measures, particularly MCC +12.7%, but AUC slightly declined. For AD disease, the feature selection marginally improved ACC and F1-score. Selecting significant features for frailty-related AD characteristics led to steady improvements in all measures, with the largest rise in MCC +11.0% showing better balanced classification performance.

Because variations in walking speed, stride length, and the number of gait events reliably reflect increasing severity of the disease, gait analysis offers a useful biomechanical window into frailty. People show slower, shorter, and fewer steps as their condition worsens, indicating a disjointed and less energy-efficient gait. This is consistent with clinical findings that frail people have decreased fine motor control and dwindling physiological reserves. While gait measures are important, they may not fully reflect the complexity of frailty, as indicated by the limited efficacy of machine learning models like logistic regression and Random Forest in identifying these gait-based phenomena.

Importantly, the data suggest that the difference in frailty in this group is due to a reduction in overall motor efficiency, not just a deceleration. We used the attributes recognized and ranked by the Random Forest algorithm, walking velocity, stride length and gait event frequency and we observed a clear increasing trend in locomotor efficiency. The most robust group was the most efficient, followed by a moderate group, and the severely frail group obtained the lowest scores on this functional dimension. It demonstrates that frailty increases in a systematic, not chaotic, manner along this continuum of biomechanical efficiency.

The fact that reduced fine gait control and slower walking are important characteristics of frailty is especially notable, suggesting that frailty manifests not only as decreased speed but also as a less stable and biomechanically demanding gait, providing fresh perspectives on the underlying causes of the condition.

The development of mild gait asymmetry is another significant observation. Analysis reveals that walking speed and turning angle frequently vary between the left and right sides, indicating that both overall gait performance and limb coordination decline with increasing frailty. Interestingly, the relative asymmetry in walking speed tends to rise contrary to the absolute difference between sides decreases. This suggests that both legs slow down together in severe frailty, but their functional relationship becomes less stable. Therefore, both reduced motor function and disturbed symmetry are indications of advanced frailty.

Further analysis of covariance structures and distribution patterns for the identified common characteristics, e.g., level of Physical Activity and Body Mass Index, showed that subjects with Alzheimer’s disease and frailty syndrome had similar symmetric deviations compared to healthy controls. This behavioral coordination of metabolic and functional measures strongly suggests that the observed overlap is a true convergence of systemic biological vulnerability, and not an artifact of the predictive transfer of the machine learning algorithm alone.

On the other hand, a closer look at the Alzheimer’s disease dataset reveals a far greater reliance on clinical and functional factors. The great performance of prediction models can be explained by the high discriminative capacity of cognitive scores, daily living activity measurements, and behavioral symptoms. Although, the prediction models help with the early diagnostics of AD, human observation should still be there, as these characteristics are direct clinical manifestations of the illness rather than just risk factors. Predictive power falls to chance levels when such signs are eliminated and the analysis is restricted to broad risk factors alone, indicating that generic premorbid factors are inadequate for capturing disease start within this dataset.

In this dataset, cognitive and functional factors account for virtually all of the ability to discriminate between affected and unaffected people. In contrast, more general risk factors such as lifestyle, metabolic or demographic characteristics have little effect on predictive power. This pattern implies that in this population, the overt clinical features of Alzheimer’s disease are more important than preclinical risk factors. The distinction between Alzheimer’s disease models and frailty models reflects an underlying biological reality rather than just technical variations. Alzheimer’s disease shows as a more advanced stage with marked cognitive and functional deterioration; whereas, frailty is an earlier, systemic disorder with abnormalities at the motor and metabolic levels. Therefore, rather than being completely distinct disease entities, these two situations can be seen as different positions along a continuum of biological vulnerability.

Gait is an important link between frailty and cognitive impairment. Research has shown that a slower gait speed is not only an indicator of frailty but it could be an early indicator of cognitive decline, which often takes place years before dementia is diagnosed [42]. This indicates that the gait abnormalities seen in frail individuals should be viewed not only as manifestations of physical dysfunction but also as potential early indicators of a neurological disorder that may subsequently present as cognitive impairment.

These observations taken together provide a conceptual framework that frailty may be a precursor and contributor of cognitive decline. Alzheimer’s disease-related cognitive decline may be preceded by problems with energy metabolism, fronto-subcortical circuits or sensory-motor integration, leading to increased step variability, slower gait and decreased motor efficiency.

It is interesting that these datasets, frailty and Alzheimer’s disease, turn out to be two aspects of a larger biological susceptibility, not two separate disorders. The main symptom of frailty is reduced motor efficiency, but reduced cognitive and functional ability is a main symptom of Alzheimer’s disease.

Including certain AD features directly connected with the frailty syndrome resulted in a net gain in predictive-classification accuracy over models based only on frailty, ACC = 0.7233, MCC = 0.3884. Further optimization by identifying critical AD traits related to frailty resulted in the best performance in this subgroup, with ACC = 0.7419, F1-score = 0.6288, and MCC = 0.4311. The findings suggest that some AD dimensions associated with frailty have a larger predictive signal than isolated frailty traits.

## 4. Limitations of Study

The limits of the study may vary from the key features determined by RF due to the parameter settings, correlated predictors or sample variability. Models trained and tested on a single dataset or homogeneous population may not generalize to other clinical settings, geographic regions, or demographic populations. A relatively small number of observations or participants may limit the generalizability of the conclusions of the study and its statistical power. In addition, machine learning models are more prone to overfitting when the dataset is small.

Since the cognitive and functional scores used as predictors are an important part of the diagnostic criteria of the pathology, the risk of circularity of the data is a major limitation of the model created for Alzheimer’s disease. The good performance of the classifier thus suggests a great diagnostic discrimination capacity of already apparent cases, rather than longitudinal prediction or early screening in preclinical phases.

## 5. Conclusions

The current study supports the notion that frailty and Alzheimer’s disease are not distinct clinical entities, but rather divergent expressions of the same process of systemic decline, anchored in a common architecture of vulnerability that manifests itself in different physiological systems.

The crux of these findings is that the discovered disease signal is mostly clinical rather than epidemiological, suggesting that traditional risk factors have low discriminatory value in this situation; whereas, functional characteristics dominate the prediction picture. Thus, frailty emerges as a phenotype of latent motor dysfunction, defined not only by a decrease in speed, but also by a complex alteration of gait efficiency, control, and symmetry, with the transition to the second severity class indicating a qualitatively deeper stage of degradation rather than a simple accumulation of deficits. In the mirror, Alzheimer’s disease appears as a phenotype of apparent cognitive-functional dysfunction, the substance of which is reflected considerably more faithfully by a cognitive burden assessment, the cognitive burden assessment, than by a traditional risk index, which seldom discriminates.

As a result, this dependency highlights the importance of functional and motor markers in early diagnosis, reinforcing the concept that clinical performance and motor organization are stronger indicators of systemic integrity than traditional epidemiological characteristics.

Overall, the results suggest that Alzheimer’s features are much more predictive, and that the inclusion or selection of Alzheimer’s features associated with frailty strengthens the complex biological relationship between the two syndromes. Within this research context, the predictive analytics are primarily a computational validation of the physiological convergence along the neuromotor axis and not an immediate practical clinical screening tool.

## Figures and Tables

**Table 1:** The values of tuning hyperparameters.

Hyperparameter	Values
Estimators number	100, 200, 300
Max depth	5, 10, 20
Minimum samples split	2, 5, 10
Minimum samples leaf	1, 2, 4

**Table 2:** Quality metrics computed from confusion matrices.

Dataset	ACC	F1-Score	MCC	AUC
Whole AD dataset	0.9465	0.9226	0.8823	0.941
Important features extracted from AD dataset	0.9488	0.9272	0.8878	0.936
Whole frailty dataset	0.6418	0.6195	0.3245	0.774
Important features of frailty	0.6567	0.6484	0.3657	0.763
Relevant features extracted from AD that are related to frailty	0.7233	0.5993	0.3884	0.822
Important features from AD what are related with frailty	0.7419	0.6288	0.4311	0.826

## Data Availability

The dataset related to geriatric frailty was analyzed from the article “Prevalence and Determinants of Frailty in the Elderly with Quantifying Functional Mobility’’. Data availability: https://doi.org/10.6084/m9.figshare.c.7874411, accessed on 15 April 2026, code availability: https://github.com/aabbasi-polsl/FRAILPOL_classification, accessed on 15 April 2026. The Alzheimer’s dementia dataset was analyzed from Kaggle, author Rabie El Kharoua, https://www.kaggle.com/datasets/rabieelkharoua/alzheimers-disease-dataset, accessed on 15 April 2026.

## Abbreviations

AI	Artificial intelligence
AUC-ROC	Area Under the Receiver Operating Characteristic Curve
MMSE	Mini-Mental State Examination
ADL	Activities of Daily Living
BMI	Body Mass Index
ID	Identity
RF	Random Forest
AD	Alzheimer’s disease
ACC	Accuracy
MCC	Matthews Correlation Coefficient
AUC	Area Under the Curve
